# A Systematic Review Looking at the Current Best Practices as well as Primary Care Practitioner's Views on the Diagnosis and Treatment of Childhood Obesity

**DOI:** 10.7759/cureus.34346

**Published:** 2023-01-29

**Authors:** Umar Ahmed, Mohammed S Mahmood, Matt Parsons, Hyatt O'callaghan, Olga Pawlik, Saif Chaudhary, Maryam Ahmed

**Affiliations:** 1 Ophthalmology, Surrey and Sussex Healthcare NHS Trust, London, GBR; 2 Hospital-Based Medicine, University Hospitals of Morecambe Bay NHS Foundation Trust, Lancaster, GBR; 3 Hospital-Based Medicine, Hampshire Hospitals NHS Foundation Trust, Hampshire, GBR; 4 Hospital-Based Medicine, Swansea Bay University Health Board, Swansea, GBR; 5 General Medicine, Nepean Hospital, Kingswood, AUS; 6 Hospital-Based Medicine, Imperial College London, London, GBR; 7 Orthopaedics and Trauma, Royal Surrey County Hospital, Guildford, GBR

**Keywords:** pediatrics & child health, treating obesity, public health care, primary care medicine, childhood obesity

## Abstract

Childhood obesity is a significant and growing issue, with the WHO recognising worldwide childhood obesity rates as an epidemic. Primary care is often the first point for monitoring a child’s development over time, hence could play an integral part in recognising and addressing childhood obesity. As a result, our systematic review has two objectives. The primary objective is to review the current evidence on best practices in diagnosing and treating childhood obesity. The secondary objective is to review recent qualitative studies looking into the view of primary care practitioners on the treatment and diagnosis of childhood obesity. The rationale for this is to help determine what opportunities there are in primary care in the NHS to tackle childhood obesity. Using searches in MEDLINE, EMBASE, PSYCHINFO, HMIC and NHS evidence over a five-year period from March 2014 to March 2019, a total of 37 studies were eligible for inclusion in the review. Out of these, 25 studies identified exploring the diagnosis and treatment of childhood obesity. A few key themes in these studies were identified, including motivational interviewing, m-health, tools and resources used in consultations, the use of dieticians in the primary care team and factors concerned with the identification of obesity in children. The rest of the 12 qualitative studies involved eliciting the views of direct stakeholders about the diagnosis and treatment of obesity in children. Eight of the studies investigated providers’ views towards the role of primary care practitioners in treating childhood obesity, two investigated the parents of obese children’s perspectives and the other two investigated general practitioners' (GPs) views towards specific tools and resources. Regarding our primary objective, our findings showed many studies looking at interventions to reduce the BMI in obese children fail to do so in a statistically significant way. However, a few interventions have been more consistent in reducing BMI and obesogenic behaviours. Those interventions include ones utilising the motivational interviewing technique and those targeting families, rather than children. Another key finding was that tools and resources available to primary care providers can significantly impact their ability to diagnose and treat obesity, particularly when looking at the detection. Finally, evidence regarding the clinical effectiveness of e-health is limited, with views on their use also mixed. Regarding our secondary objective, the qualitative research identified demonstrated many common views from GPs across different countries. It showed healthcare providers (HCPs) perceiving the parents as lacking in motivation to address the issue, HCPs not wanting to damage the relationship with their patients due to it being a sensitive topic to bring up, and a lack of time, training and confidence. However, some of these views may not be generalisable to the UK due to cultural and system differences.

## Introduction and background

Definitions of obesity have changed over time; nonetheless, they can be uniformly defined as an excess of body fat. The National Child Measurement Program (NCMP), which measures the height and weight of UK children in Reception class and Year 6, defines childhood obesity as being the 95th percentile or higher of the British 1990 growth reference and overweight as the 85th percentile of that reference or higher [1].

Childhood obesity is a significant and growing issue. Halting and reversing childhood obesity rates has become a priority for governments and health institutions across the world. The WHO recognised worldwide childhood obesity rates as an epidemic and the World Health Assembly has adopted the “Global Action Plan”, agreeing to reach the target of halting global obesity rates by 2025 [2].

An increased BMI in children is associated with comorbid conditions such as attention-deficit/hyperactivity disorder (ADHD), depression, developmental delay, asthma, allergies, headaches and ear infections. Obese children are up to two times more likely to have multiple related comorbidities compared to children with a healthy weight. Furthermore, children with obesity immediately have a higher risk parameter for cardiovascular disease like raised blood pressure and cholesterol levels and significantly raised fasting insulin levels, a known marker for diabetes [3].

Beyond immediate and future health consequences, obesity in childhood is associated with a myriad of socio-emotional consequences. A systematic literature review (SLR) of 53 studies conducted between 2006 and 2013 showed a significantly higher likelihood of psychopathology in obese children. These studies show obese young people have a higher prevalence of eating disorders such as bulimia. In girls, there is also a linear relationship between body dissatisfaction and increasing BMI whereas for boys the relationship follows a U shape with dissatisfaction levels increasing at both extremes. The development of obesity during adolescence was shown to be related to psychosocial distress, low mood, poor self-esteem and social anxiety. Research confirms the clear negative impact of obesity on a child’s self-worth and self-competence compared to healthy-weight children. In young girls, the likelihood of developing depression has a positive correlation with increasing BMI [4].

Although most medical consequences are largely reversible with weight loss, many health and psychological consequences continue to impact the quality of life [5].

The UK Prime Minister decided to make tackling childhood obesity a goal for this Government, working with the Secretary of State for Health and Social Care to make improving children’s health a critical part of the long-term plan for the NHS through the publishing of the childhood obesity plan in 2016 [6].

The British Journal of General Practice has recognised that general practitioners (GPs) should play an integral role in identifying and addressing childhood obesity [7]. GPs are often the first point of contact for parents and children and monitor a child’s development over time, therefore theoretically have a high chance of identifying children on the path to becoming obese and intervening. Identification of a method that allows GPs to combine the individual small successes of previously tested interventions could theoretically magnify their effect and catalyse their employment on a national scale [8].

As a result, this systematic review has two objectives. The primary objective is to review the current evidence on best practices in the diagnosis and treatment of childhood obesity. The secondary objective is to review recent qualitative studies exploring the role of primary care providers in diagnosing and treating childhood obesity. The rationale for this is to help determine what opportunities there are in primary care in the NHS to tackle childhood obesity.

## Review

Data sources and search methods

This systematic review will evaluate multiple literature databases in accordance with PRISMA (Preferred Reporting Items for Systematic review and Meta-Analysis Protocols) guidelines. Searches were run in MEDLINE, EMBASE, PSYCHINFO, HMIC (Health Management Information Consortium) and NHS evidence, covering a five-year period from March 2014 to March 2019. Search terms included were (childhood OR adolescent OR infant OR children OR child) AND (obes* OR overweight) AND (primary care OR GP OR general pract* OR family medicine).

Inclusion and exclusion criteria

Research articles will be included if they satisfy the following inclusion criteria as demonstrated in Table 1 and Table 2. Articles excluded include non-English language papers and editorials.

**Table 1:** Primary inclusion criteria - The current evidence on best practices in the diagnosis and treatment of childhood obesity

Primary inclusion criteria	
Population, participants or conditions of interest	Children (ages 2-18), any gender, overweight or obese BMI. Population NOT restricted to the UK
Interventions or exposures	People who are overweight or obese and have been seen in a primary care setting and undergone an intervention to reduce their BMI
Comparisons or control groups	People who have been seen in a primary care setting and not undergone an intervention to reduce their BMI
Outcomes of interest	Effectiveness of primary care interventions, number of overweight or obese children undertaking interventions, reasons why interventions were not undertaken or were not effective. Attitudes of parents, children and physicians towards the interventions
Setting	Primary care
Study designs	Randomised control trial (RCT), qualitative analysis, cross sectional study, systematic review, case control.
Search period	01/03/2014 - 01/03/2019

**Table 2:** Secondary inclusion criteria - Exploring the role of primary care providers in diagnosing and treating childhood obesity

Secondary inclusion criteria	
Population, participants or conditions of interest	Children (ages 2-18), any gender, overweight or obese BMI. Population NOT restricted to the UK. OR parents of said children. OR primary care providers
Interventions or exposures	No intervention necessary
Outcomes of interest	Qualitative studies exploring the role of primary care providers in diagnosing and treating childhood obesity
Setting	Primary care
Study designs	Qualitative study design e.g.: interview surveys and focus groups
Search period	01/03/2014 - 01/03/2019

Screening and data extraction

Research articles were initially identified via the search strategy previously discussed, with duplicate articles removed. Using a shared, predesigned Microsoft Excel form, two reviewers then reviewed articles based on title and subsequently by abstract. Disagreements were resolved via discussion between the two authors, with the articles being included in a full-text review if a consensus could not be agreed. Full-text articles were then reviewed again by two authors, with a failure to agree on the inclusion of an article resolved by the involvement of a third author. The full-text articles were subsequently grouped regarding whether they relate to the primary objective or secondary object. Key themes were identified regarding the primary objective through discussion amongst the three reviewers of the full-text articles. The full-text articles regarding the secondary objective were reviewed depending on the type of stakeholder views elicited, whether they were the views of parents of obese children or primary care practitioners.

Risk of bias analysis

The included studies had undergone a risk of bias analysis using the Joanna Briggs Institute’s Critical Appraisal tools for each appropriate study type [9]. These tools were used independently by two authors, with any disputes resolved via discussion. If this was not successful a third author was involved to resolve disputes. The risk of bias of each article was then deemed to be either high, moderate or low depending on the percentage of items on the relevant checklists that were satisfied, with a high risk of bias deemed less than or equal to 49%, moderate 50% to 69% and low risk greater or equal to 70% of checklist items satisfied. This allowed us to assess the quality of the articles included.

Results

**Figure 1:**
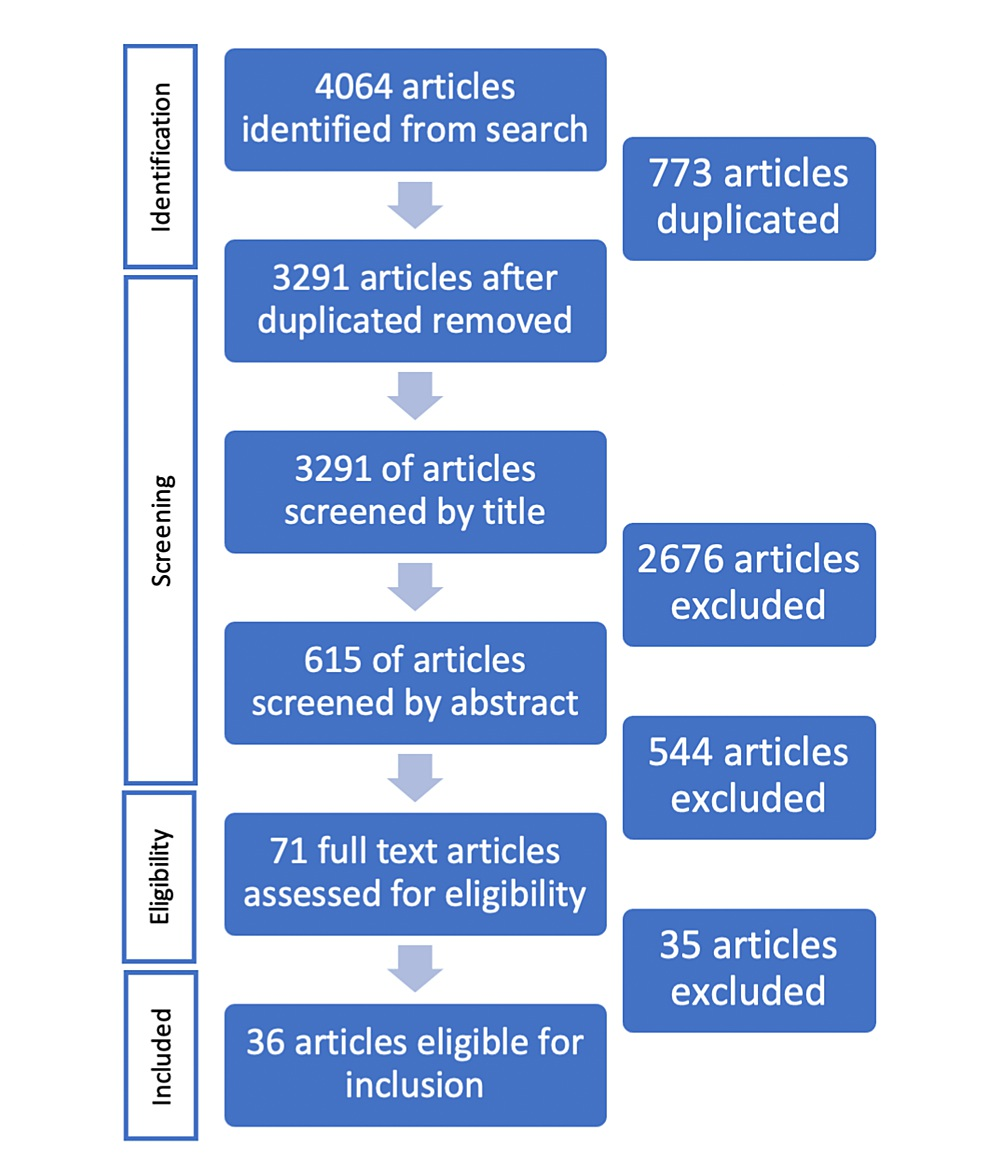
PRISMA diagram - Articles identified in each phase of literature review screening

Initially, 4064 articles were identified in the search, with 773 removed as duplicates. In the first phase, the articles were screened by title, removing 2676 based on the inclusion criteria. Secondly, the remaining articles were screened by abstract, removing 544. Finally, the full text of the articles was read and assessed for eligibility in line with the inclusion criteria. This left us with 36 studies eligible for inclusion in the review (See Figure 1). Of these studies included, none of which were classified as high risk of bias, with all articles satisfying either low or moderate risk of bias.

Discussion

Due to the two distinct aims of the literature review, the results will be discussed in two separate sections; Evidence for the diagnosis and treatment of childhood obesity and Qualitative evidence demonstrating direct stakeholder views on the diagnosis and treatment of childhood obesity.

Evidence for the Effective Diagnosis and Treatment of Childhood Obesity

Twenty-four studies identified exploring the diagnosis and treatment of childhood obesity. A few key themes in these studies were identified, including motivational interviewing, m-health, tools and resources used in consultations, the use of dieticians in the primary care team and factors concerned with the identification of obesity in children.

1) Motivational interviewing: Three randomised control trials (RCT) were identified directly comparing the effects of motivational interviewing (MI) with a control group. MI can be described in brief as eliciting information from the patient, providing non-judgemental information and eliciting the patient’s understanding [10]. Of the other two studies, it was found to be an effective technique for Registered Dieticians to use in primary care and had a high degree of feasibility on a larger scale, including good cost-effectiveness [11,12].^ ^Overall, all three studies demonstrated a statistically significant decrease in BMI when using the motivational technique compared to the control which used the standard technique in each case. The studies provide evidence to use the technique in routine practice with overweight and obese children. An interesting study observed that physicians used MI inconsistent techniques twice as often and MI consistent techniques when discussing weight with adolescents and when inconsistent techniques were used, it lengthened encounter times [13]. This demonstrates that despite evidence to show its effectiveness, physicians may not be using the technique in routine practice (See Table 3).

**Table 3:** Evidence for the effective diagnosis and treatment of childhood obesity - Motivational interviewing

Reference	Year of publication	Country	Journal	Study Design
Small et al. [10]	2014	USA	Journal of Pediatric Health Care	Randomised Control Trial
Resnicow et al. [11]	2015	USA	Pediatrics	Randomised Control Trial
Smith et al. [12]	2018	USA	Implementation Science	Randomised Control Trial
Pollak et al. [13]	2014	USA	Patient Education Counseling	Observational Study

2) E-health: Technology has the potential to play an even greater role in medicine and can act as a catalyst to help patients take more responsibility for their health. Two studies were identified that directly explored the use of technology to help treat obesity. In an RCT, it was concluded that a novel m-health intervention delivered on a tablet has the potential to encourage parents of unhealthy-weight children to uptake healthier behaviours [14]. Another study explored the feasibility of an integrated care model utilizing personalized messaging and teleconsultations to augment standard practices and showed significant reductions in the children’s BMI [15]. However overall evidence for the effectiveness of E-health is limited (See Table 4).

**Table 4:** Evidence for the effective diagnosis and treatment of childhood obesity - E-health

Reference	Year of publication	Country	Journal	Study Design
Byrne et al. [14]	2018	Canada	Pediatric Obesity	Randomised Control Trial
Fleischman et al. [15]	2016	USA	Clinical Obesity	Randomised Pilot Study

3) Identification of obesity in children: It is obvious to state that the identification of obese children is fundamental to the process of treating obesity promptly. One study showed that a staff training intervention significantly increased the accuracy of overweight and obese coding on an electronic health record system, demonstrating the importance of training systems [16]. A second study utilized pictures of children to assess the accuracy of clinical assessment compared to measurement and concluded that there is a statistically significant disparity between visual judgement and physical measurement of BMI [17]. A cross-sectional study demonstrated that fewer than one-fifth of children identified as obese by the National Child Measurement Scheme in Hackney, London, consult a GP about their weight (See Table 5) [18].

**Table 5:** Evidence for the effective diagnosis and treatment of childhood obesity - Identification of obesity in children

Reference	Year of publication	Country	Journal	Study Design
Camp et al. [16]	2019	USA	Journal of Pediatric Health Care	Experimental Study
Gohil et al. [17]	2017	USA	Global Pediatric Health	Cross-sectional study
Dezateux et al. [18]	2017	USA	Lancet	Cross-sectional study

4) Tools and resources: Three studies evaluated tools and resources available to primary care providers in treating childhood obesity, rather than direct interventions. Firstly, a large quality improvement project in Chicago, Illinois attempted to implement the Chronic Care Model to improve provider identification, prevention, and management of childhood obesity [19]. The study made significant changes to the provider’s Electronic Medical Record system, providing decision support tools and better options on the drop-down menus. The results included a significant increase in primary care provider adherence to expert guidelines. A second quality improvement project implemented a tracking form to encourage providers to have more comprehensive discussions on preventative care visits and utilise the motivational interviewing technique [20]. This change resulted in significant increases in the usage of the technique and more questions being asked across all aspects of obesity as governed by expert guidelines. Finally, an RCT explored the implications of giving a novel-written Wellness Action Plan to carers of obese children during visits [21]. The plan resulted in better recall of the agreed plan and improved adherence to it. There is reasonable evidence to show that many tools and resources could significantly improve the diagnosis and treatment of obesity (See Table 6).

**Table 6:** Evidence for the effective diagnosis and treatment of childhood obesity - Tools and resources

Reference	Year of publication	Country	Journal	Study Design
Cygan et al. [19]	2018	USA	Clinical Pediatrics	Quality Improvement Project
Rankin et al. [20]	2015	USA	International Journal of Pediatrics and Adolescent Medicine	Quality Improvement Project
Kharofa et al. [21]	2015	USA	Preventive Medicine Reports	Randomised Control Trial

5) Interventions aimed at reducing BMI: Out of the remaining 12 studies, all are based on interventions aimed at reducing BMI in overweight and obese children. Firstly, the first five are RCTs and two of which demonstrated that even low-intensity interventions with minimal contact time can have a significant effect on reducing obesogenic behaviours and reducing BMI [22,23]. Three of the other studies demonstrated that a more intensive, multifaceted approach can have a profound effect on obesogenic behaviours. The first of which demonstrated the effectiveness of combining behavioural strategy sessions, weekend activities, supervised exercise and group sessions with the carers of the children [24]. In particular, this approach had a very high adherence rate with few sessions being missed. The second study used a multifaceted approach encompassing the motivational interviewing technique and educational videos and demonstrated promising changes in behaviour, however, the effects at two years were not significant [25]. Multidisciplinary teams consisting of dieticians, physiotherapists, nurses and GPs have also been proven to be effective, however, there was no difference in changes in BMI or obesogenic behaviours between groups with different professionals delivering the intervention [26].

The remaining seven studies were pilot trials and quality improvement projects. Two such studies focused on low-intensity interventions, both showing a degree of improvement in BMI and obesogenic behaviours. One of which focused on weekly goal setting, and the other took a multidisciplinary approach involving dieticians in the process along with GPs [27,28]. A third study also showed reductions in BMI z-score and percentile when taking a family goal-setting approach [29]. Interestingly, they also incorporated some MI into their intervention. Three of the remaining four studies took a multidisciplinary, collaborative approach incorporating multiple techniques. The first of which demonstrated significantly improved attendance to lifestyle modification visits when a clinic-community approach was adopted [30]. The second demonstrated some degree of effectiveness in running a multidisciplinary clinic [31]. Some degree of effectiveness was demonstrated with family group sessions targeting social support, mind-body exercises, physical activity and nutrition, however, the effects did not persist for two years after the interventions [32]. Finally, an interesting paper demonstrated the short-term efficacy and feasibility of implementing culturally targeted obesity interventions (See Table 7) [33].

**Table 7:** Evidence for the effective diagnosis and treatment of childhood obesity - Interventions aimed at reducing BMI

Reference	Year of publication	Country	Journal	Study Design
Wylie-Rosett et al. [22]	2014	USA	Journal of Pediatric Health Care	Randomised Control Trial
Looney and Raynor [23]	2015	USA	Pediatrics	Randomised Control Trial
Serra-Paya et al. [24]	2018	USA	Implementation Science	Randomised Control Trial
Rifas-Shiman et al. [25]	2014	USA	Patient Education Counseling	Observational Study
Forsell et al. [26]	2019	Sweden	Acta Paediatrica	Randomised Control Trial
Denney-Wilson et al. [27]	2014	Australia	Journal of Paediatrics and Child Health	Randomised Control Trial
Tucker et al. [28]	2019	USA	Health Behaviors, and Body Mass Index. Nutrients	Randomised Control Trial
Jortberg et al. [29]	2016	USA	The Journal of the American Board of Family Medicine	Randomised Control Trial
Tripicchio et al. [30]	2018	USA	Childhood Obesity	Randomised Control Trial
Sauven et al. [31]	2014	UK	Archives of disease in childhood	Audit
Bottino et al. [32]	2017	USA	Clinical Pediatrics	Quality Improvement Project
Chen et al. [33]	2015	USA	Journal of Pediatric Nursing	Quality Improvement Project

Qualitative Studies Involved Eliciting the Views of Direct Stakeholders About the Diagnosis and Treatment of Obesity in Children

The literature review identified 12 qualitative studies eligible for inclusion; one from Turkey, one from the Netherlands, three from the UK and seven from the USA. Geographical location is important to consider when looking at these studies since perceptions and systems vary greatly from region to region, therefore the findings will have limited generalisability to the UK. Eight of the studies looked into provider’s views towards the role of primary care practitioners in treating childhood obesity, two looked into the parents of obese children’s perspectives and the other two looked into GP’s views towards specific tools and resources.

1) The views of parents of obese children toward the role of primary care in their treatment: The two studies based in the US elicited those parents of obese children perceived the main roles of GPs in their care to be: monitoring weight, providing guidance about weight loss, using discretion when bringing up the sensitive issue and they wanted GPs to emphasise incremental routine changes, rather than radical ones and suggest healthy lifestyle choices, rather than specific weight loss diets [34]. A second study elicited that parents of obese children perceived several barriers to accessing care, including the clerical referral process, time and cost constraints, location and schedule and child motivation [35]. Parents suggested that to improve access, there should be a systematic referral process whereby they can easily gain access to the service, the programmes should be organised closer to where they live and at more convenient times and they should be free to access. However interesting the results, it is important to consider that the healthcare structure in the USA is vastly different from the NHS, therefore barriers to access may be different, for example, cost and societal differences may lead to a discrepancy in the perceived role of healthcare providers (See Table 8).

**Table 8:** Evidence of the views of parents of obese children toward the role of primary care in their treatment

Reference	Year of publication	Country	Journal	Study Design
Turer et al. [34]	2016	USA	Maternal & Child Nutrition	Qualitative Study
Kulik et al. [35]	2017	USA	Journal of Child Health Care	Qualitative Study

2) Primary care practitioners’ perceptions of the role of primary care in preventing and treating obesity in children: Eight studies were identified that assessed primary care providers' views of their role in treating childhood obesity. Out of all the studies, a few issues were brought up by GPs in all countries. This includes: healthcare providers (HCPs) perceiving the parents as lacking in motivation to address the issue, HCPs not wanting to damage the relationship with their patients due to it being a sensitive topic to bring up, and a lack of time, training and confidence [36-38]. One study based in Turkey brought up the issue that GPs didn’t feel as if measuring children ages 5-15 was important [39]. A separate study in the USA identified a gap in care for children aged 18 months to 5 years [40]. Similarly, a UK-based study identified that GPs felt that contact with children was inconsistent and that having obese children not present to the clinic for long periods is an issue in identifying obesity [41]. A second UK-based study elicited that GPs had concerns over the usefulness of the National Child Measurement Scheme [42]. It was suggested that patients had a psychological reactance to receiving a “fat letter” and it fostered hostility and resentment towards healthcare professionals and was acting as a barrier to the treatment of obese children. Finally, a USA-based study had GPs suggesting that case managers and health coaches should be implemented a team-based approach to treat obesity [43].

The final two studies investigated perceptions of tools and resources available to GPs. The first one assessed which tools GPs were using, however, it was conducted in Canada and the tools and systems available are different from those used within the NHS, however, they did conclude that developers of these tools should work with primary care providers to better fit their needs and the needs of their patients [44]. The importance of tools and resources is emphasised previously in this literature review. The final study looks into GPs’ perceptions towards the Well Child Visits conducted in the USA and concludes that it is important to discuss obesity in Well Child Visits each year [45]. This has limited generalisability to the NHS since we do not have Well Child visits, however, we do have regular visits with children, for example, the vaccination scheme where there are opportunities to discuss weight and it could be important to elicit whether GPs think there should be a regular meeting with every child. This would be in line with other literature analysed earlier stating there are gaps in children’s care between certain ages and low, inconsistent presence at the clinic is a barrier to identifying obesity in children (See Table 9).

**Table 9:** Evidence of primary care practitioners’ perceptions of the role of primary care in preventing and treating obesity in children

Reference	Year of publication	Country	Journal	Study Design
Fildes et al. [36]	2017	UK	Lancet	Qualitative Study
Schalkwijk et al. [37]	2016	Netherlands	BMC Health Services Research	Qualitative Study
Busch et al. [38]	2018	USA	Journal of Pediatric Health Care	Qualitative Study
Sakarya et al. [39]	2018	Turkey	European Journal of General Practise	Qualitative Study
Bourgeois et al. [40]	2016	Canada	CMAJ Open	Qualitative Study
O’Donnell et al. [41]	2017	UK	JRSM Open	Qualitative Study
Johnson et al. [42]	2018	UK	BMC Research Notes	Qualitative Study
Rhee et al. [43]	2018	USA	BMC Health Services Research	Qualitative Study
Avis et al. [44]	2016	Canada	Patient Education and Counselling	Qualitative Study
Bonnet et al. [45]	2014	USA	Journal of Obesity	Qualitative Study

Limitations of systematic literature review

Despite the strengths of this study it's important to mention the limitations experienced. When writing up the findings of the review, limited details of each study were included for the sake of accessibility and clarity, therefore it is difficult to compare the quality of each evidence point and understand which ones should be weighted higher than others. Similarly, due to the variety of study types, a standard quality assessment was not used for each article, therefore making it difficult to compare each study in terms of quality. Finally, our search criteria excluded non-English published articles which could have been used to create a better understanding of our research objectives.

## Conclusions

Interventions with the strongest evidence base include ones utilising the motivational interviewing technique and those targeting families, rather than children. The motivational interviewing technique was shown to be efficacious and was studied widely in the past five years. It can also be seen that group-based sessions have a higher adherence rate than individual clinics and patients report higher satisfaction. The non-interventional studies demonstrated a few key findings. One of which was that tools and resources available to primary care providers can have a significant impact on their ability to diagnose and treat obesity, particularly when looking at detection. Also, there is a small amount of evidence demonstrating the potential of e-health, however, the evidence of clinical effectiveness is limited. The qualitative research identified demonstrated many common views from GPs across different countries, however, some of these views may not be generalisable to the UK due to cultural and systems differences. In addition, information was lacking on how GPs felt the system could be improved to enable them to treat obesity more effectively and view towards navigating the NHS system.

Through comparison of the findings of these two distinct primary objectives, it allows the interpretation of which of the best-evidenced interventions in managing childhood obesity would be most compatible with primary care. It allows better understanding of the barriers to implementing interventions into primary care, which can allow future interventions to adapt their strategies in accordance with primary care views.
